# Navitoclax Most Promising BH3 Mimetic for Combination Therapy in Hodgkin Lymphoma

**DOI:** 10.3390/ijms232213751

**Published:** 2022-11-09

**Authors:** Myra Langendonk, Nienke A. M. Smit, Wouter Plattel, Arjan Diepstra, Tom van Meerten, Lydia Visser

**Affiliations:** 1 Department of Hematology, University Medical Center Groningen, University of Groningen, 9713 GZ Groningen, The Netherlands; 2 Department of Pathology and Medical Biology, University Medical Center Groningen, University of Groningen, 9713 GZ Groningen, The Netherlands

**Keywords:** Hodgkin lymphoma, anti-apoptotic proteins, BH3 profiling, therapy

## Abstract

The treatment of young patients with Hodgkin lymphoma (HL) is often successful but a significant proportion of patients suffers from late toxicity. In the current era there are new opportunities for less toxic and more targeted treatment options. In this respect, the anti-apoptotic pathway is an attractive target since Hodgkin tumor cells abundantly express components of this pathway. We measured the effect of BH3 mimetics that interfere with anti-apoptotic proteins in cell lines, also in combination with the standard of care chemotherapeutic doxorubicin and the recently discovered preclinically active tamoxifen. Several anti-apoptotic BCL-2 family proteins were expressed in each case (*n* = 84) and in HL cell lines (*n* = 5). Cell lines were checked for sensitivity to BH3 mimetics by BH3 profiling and metabolic assays and monotherapy was only partially successful. Doxorubicin was synergistic with a BCL-XL inhibitor and BCL2/XL/W inhibitor navitoclax. Tamoxifen that targets the estrogen receptor β present in the mitochondria of the cell lines, could induce cell death, and was synergistic with several BH3 mimetics including/as well as navitoclax. In conclusion, targeting the anti-apoptotic pathway by the triple inhibitor navitoclax in combination with doxorubicin or tamoxifen is a promising treatment strategy in HL.

## 1. Introduction

Hodgkin lymphoma has a very good prognosis with cure rates of more than 85% with current chemotherapybased regimens for young patients. However, since many patients are in a young age group (15–35 years) late side effects of treatment are a serious problem and current research is focusing on less toxic and more specific treatment alternatives to improve long term survival of these patients.

One of the hallmarks of the tumor cells in HL is the escape from apoptosis. The expression of several anti-apoptotic proteins has been described, and with the expression of BCL-2 predicting poor outcome [1], the treatment with BH3 mimetics is an obvious option. The expression of BCL-2, BCL-XL [2], BCL-W [3] and MCL-1 [2] and also the other pro-apoptotic integral BCL-2 family proteins involved in apoptosis as BAX and BAK have been described individually in the Hodgkin Reed Sternberg (HRS) cells, but multiple anti-apoptotic proteins have not been studied in single cases [2,4]. It looks like these cells have all the ingredients to go into apoptosis but are protected by the extensive expression of multiple anti-apoptotic proteins.

The treatment of patients with obatoclax mesylate, a specific BCL-2 inhibitor, was not successful and was stopped [5]. Therefore, it seems that inhibition of just one anti-apoptotic protein is not sufficient. It has indeed been shown that also BCL-W plays an important role in the survival of HRS cells, and knock-down of not only BCL-2 and BCL-W, but also BCL-XL was necessary to induce spontaneous apoptosis in cell lines U-HO-1, KM-H2 and L428 [3]. Another possibility is that BH3 mimetics need to be combined with other apoptosis inducing agents to be more effective. In cell lines the combination of obatoclax or ABT-737 (a BCL-2/XL/W inhibitor) showed synergism with HDAC inhibitor entinostat [6], and ABT-737 could also be combined with doxorubicin and bortezomib [7]. Navitoclax, another inhibitor of BCL-2/XL/W was also successful in the combination with brentuximab vedotin and ruxolitinib in cell lines, and a mouse model with cell line HDLM2 [8].

Recently we have shown that B-cells, specifically diffuse large B cell lymphoma (DLBCL) cells, express estrogen receptor β (ERβ) that is located in the mitochondria and functions as protection against apoptosis. Treatment with the ERβ inhibitor tamoxifen can cause apoptosis in these DLBCL cells [9]. It has been shown that the ERβ is also expressed in HL cell lines [10] and HRS cells [11]. Treatment with ERβ agonist DPN inhibits growth of HL cell lines and causes autophagy (12). Our goal in this study is to check HL patients for the expression of BCL-2, BCL-XL, BCL-W and MCL-1, check HL cell lines for the sensitivity to BH3 mimetics, and check if combination therapy with doxorubicin, as a first line treatment in HL, or tamoxifen is effective and if the effect can be predicted with dynamic BH3 profiling.

## 2. Results

### 2.1. Anti-Apoptotic Proteins Are Widely Expressed by Tumor Cells in HL Tissue

**Figure 1:**
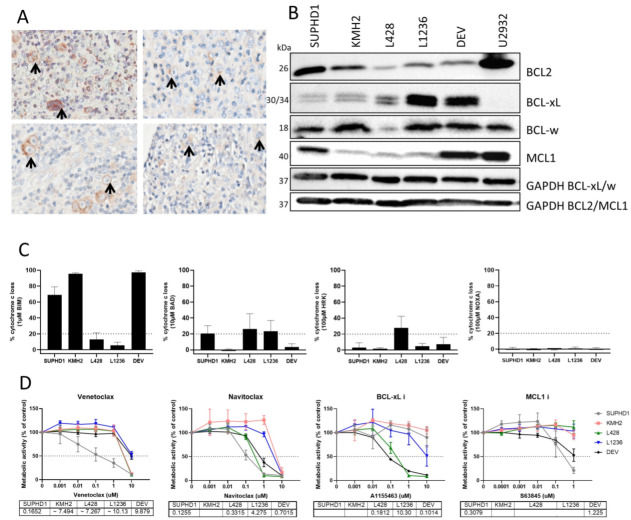
Anti-apoptotic proteins in Hodgkin lymphoma. (**A**). Immunohistochemistry for BCL-2 (top left), BCL-XL (top right), BCL-W (bottom left) and MCL-1 (bottom right) on HL cases. (**B**). Western blot of HL cell lines for BCL-2, BCL-XL, BCL-W and MCL-1. (**C**). BH3 profiles for HL cell lines for 1 µM BIM, 10 µM BAD, 100 µM HRK and 100 µM NOXA. (**D**). BH3 mimetics sensitivity for HL cell lines for venetoclax (BCL-2i), navitoclax (BCL-2/XL/Wi), A1155463 (BCL-XLi), and S63845 (MCL-1i). Sensitivity is measured by metabolic activity and normalized to untreated cells, IC50 values are given in µM if possible to calculate.

In 84 HL patients, expression of BCL-2, BCL-XL, BCL-W, and MCL-1 was tested by immunohistochemistry on TMA (Table 1, Figure 1A). In 54% of patients BCL-2 was expressed in the tumor cells, while BCL-XL and BCL-W were expressed in 88 and 90% and MCL1 in 78%. Interestingly, 60% of cHL cases were positive for BCL-2, while all 9 NLPHL cases were negative. For BCL-XL, BCL-W, and MCL-1 expression was similar in cHL and NLPHL. There were 2 patients in which we could not find expression of anti-apoptotic proteins, in one case the BCL-W, and in the other, BCL-XL and MCL-1 were not evaluable. There were 3 patients only expressing BCL-W, the remainder of the patients expressed 2 (27%), 3 (39%), or 4 (29% of cases) anti-apoptotic proteins.

### 2.2. HL Cell Lines Are Sensitive to BH3 Mimetics

The expression of the anti-apoptotic proteins was tested on 5 HL cell lines in comparison with DLBCL cell line U2932, by western blot (Figure 1B). SUPHD1 expressed BCL-2, BCL-W, and MCL-1, KMH2 expressed BCL-2 and BCL-W, L428 only expressed BCL-XL at relatively low levels. L1236 expressed mostly BCL-XL and BCL-W, while DEV expressed BCL-XL, BCL-W, and MCL-1. The cell lines showed a similar profile as the patients in their expression of anti-apoptotic proteins. Static BH3 profiling of the HL cell lines showed a strong response to BIM for SUPHD1, KMH2, and DEV, and a weak response for L428 and L1236. The response to BAD was strong for SUPHD1, L428, and L1236, and weak for DEV. A HRK response was seen for L428, and weak responses for SUPHD1, L1236, DEV, and no responses were seen for NOXA (Figure 1C). As BAD response would predict a dependency on BCL2/XL/W and HRK to BCL-XL, it would be expected that L428 is dependent on BCL-XL, while SUPHD1 depends on BCL-2 or BCL-W. L1236 and DEV are possibly sensitive to BCL-XL although there was only a weak response in HRK. For KMH2, all responses were low, and no clear prediction could be made. To validate these patterns, we checked the sensitivities of these cell lines for BH3 mimetics venetoclax (BCL-2i), navitoclax (BCL2/XL/Wi), A1155463 (BCL-XLi), and S63845 (MCL-1i) (Figure 1D). SUPHD1 was the only cell line that responded to venetoclax. SUPHD1, L428, and DEV were sensitive to navitoclax, while L428 and DEV were sensitive for the BCL-XLi (Table 2). No cell lines were sensitive to the MCL-1i. SUPHD1 is dependent on BCL-2, L428 and DEV are dependent on BCL-XL and KMH2, and L1236 are not dependent. The dependency of DEV on BCL-XL and the non-dependency of L1236 can probably be explained by the response to BIM. DEV can go into apoptosis easily as was shown by the strong BIM response, while L1236 is not since the BIM response was low. This showed that BH3 profiling was in concordance with the BH3 mimetics response for three out of five cell lines.

### 2.3. Tamoxifen Binds to Mitochondria in HL Cell Lines

**Figure 2:**
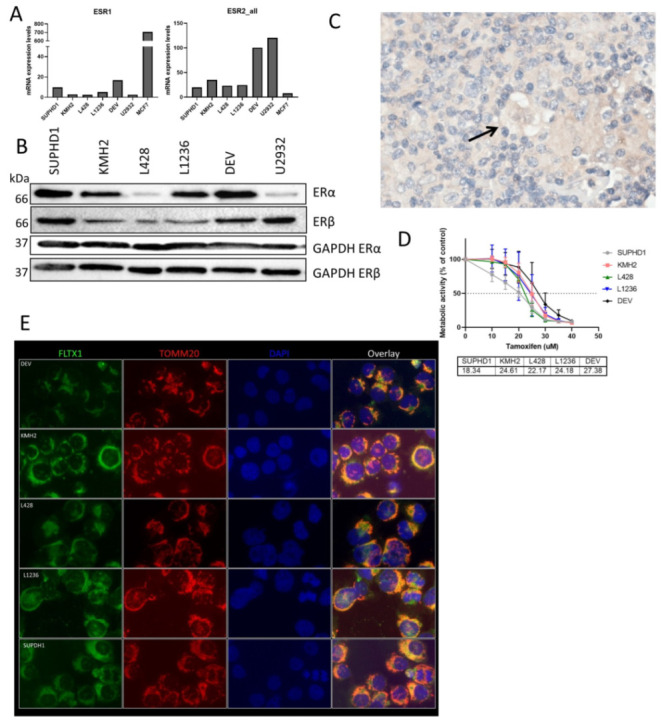
Estrogen receptor β expression in HL. (**A**). mRNA expression of ESR1 and ESR2 in HL cell lines. (**B**). Protein expression of ERα and ERβ in HL cell lines by western blot. (**C**). Immunohistochemistry for ERβ in a HRS cell in HL tissue. (**D**). Sensitivity for tamoxifen in HL cell lines. Sensitivity is measured by metabolic activity, IC50 values are given in µM. (**E**). Immunofluorescent staining of fluorescent labelled tamoxifen (FLTX1), mitochondria (TOMM20), nuclei (DAPI), and overlays on the 5 HL cell lines.

Next, we checked the expression of ER in HL. mRNA levels were low for ESR1 compared to breast cancer cell line MCF7 in the HL cell lines. For ESR2, although at lower levels than in DLBCL cell line U2932, mRNA was present in the cell lines, with the highest level in DEV (Figure 2A). ERβ was present at a protein level in these cell lines as was shown by western blotting (Figure 2B) with highest levels in SUPHD1 and DEV, levels of ERα were clearly present in SUPHD1, KMH2, L1236 and DEV. We also stained the TMA for ERβ expression, and found (low) cytoplasmic expression of ERβ in 63% of cases in the HRS cells (Figure 2C and Table 1). DPN as a specific ERβ agonist has been tested in HL cell lines before [12], but here we tested tamoxifen as a clinically available inhibitor of ER. As shown in Figure 2B, all cell lines were sensitive to tamoxifen, albeit at relatively high IC 50’s compared to DLBCL [9], ranging from 18 µM in SUPHD1 to 27 µM in DEV (Figure 2D). To show that tamoxifen is bound to the ER in the mitochondria, we incubated the cells with fluorescent labeled tamoxifen (FLTX1). We demonstrated that by using TOMM20, an antibody specifically for labelling the mitochondria, tamoxifen co-localized at the mitochondria. This suggests that tamoxifen can induce apoptosis, as we have shown in DLBCL [9].

### 2.4. Doxorubicin Induces a BCL-XL Response

**Figure 3:**
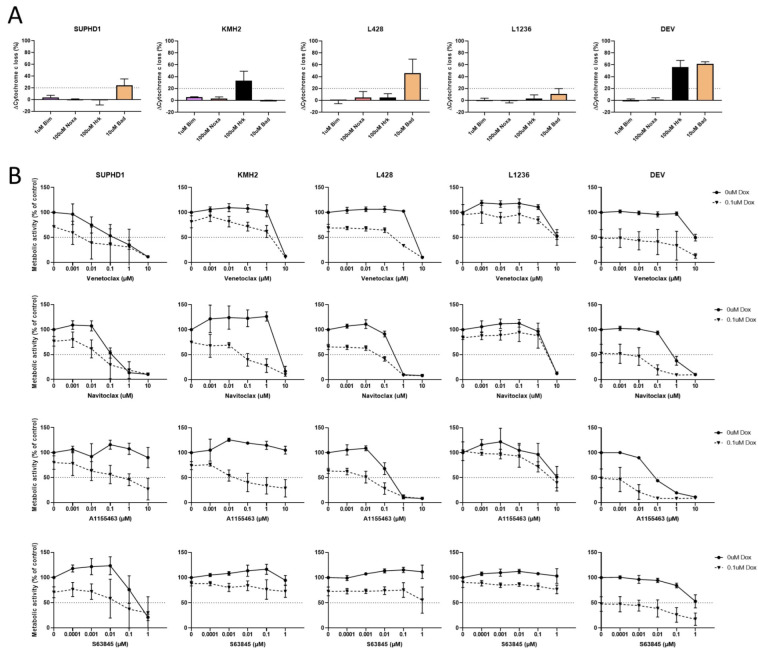
Combination therapy with doxorubicin and BH3 mimetics in HL cell lines. (**A**). Dynamic BH3 profiles for HL cell lines after doxorubicin treatment. ΔMOMP % against 1 µM BIM, 100 µM NOXA, 100 µM HRK, and 10 µM BAD. (**B**). Combination therapy of 0.1 µM doxorubicin with venetoclax, navitoclax, A1155463, and S63845 in HL cell lines. Results are given as metabolic activity normalized to untreated cells.

We started with the combination with doxorubicin as it was already shown to be synergistic with venetoclax [13] and is used in the first-line treatment of HL. IC50 curves for the cell lines in response to doxorubicin are shown in Supplementary Figure S1. Except for L1236 with an IC50 of 4 µM, all cell lines were sensitive to doxorubicin with IC50’s between 0.1 and 0.4 µM. The dynamic BH3 profiles after doxorubicin treatment showed a response in BAD for SUPHD1, L428 and DEV, HRK for KMH2 and DEV, and no response for L1236 (Figure 3A). Combination therapy (Figure 3B and Supplemental Table S1) showed an effect of the BCL-XLi for SUPHD1, KMH2, and DEV. L428 showed the strongest effect of venetoclax. In L1236, there was no effect of the BH3 mimetics, probably since there was no reaction to doxorubicin. Therefore, in general, doxorubicin induced a BCL-XL or BCL-2 response in all 5 cell lines, synergistic or additive (Table 3).

### 2.5. Tamoxifen Induces a Mixed Response in HL Cell Lines

**Figure 4:**
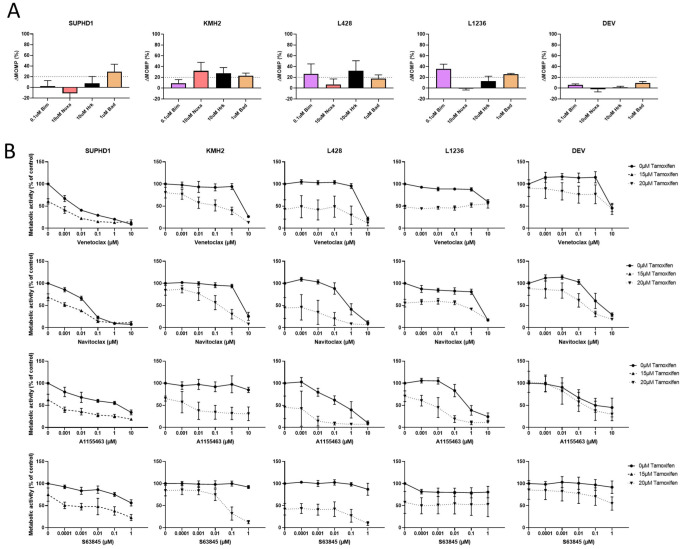
Combination therapy with tamoxifen and BH3 mimetics in HL cell lines. (**A**). Dynamic BH3 profiles for HL cell lines after tamoxifen treatment. ΔMOMP % against 0.1 µM BIM, 10 µM NOXA, 10 µM HRK and 1 µM BAD. (**B**). Combination therapy of tamoxifen with venetoclax, navitoclax, A1155463, and S63845 in HL cell lines. Results are given as metabolic activity normalized to untreated cells.

Next, we investigated the combinational effect of tamoxifen and apoptosis inhibitors. SUPHD1 was the most sensitive to tamoxifen and was treated with 15 µM tamoxifen, while the other cell lines were treated with 20 µM. For DEV, there was limited effect of tamoxifen. In the dynamic BH3 profiling after tamoxifen treatment, SUPHD1 showed a BAD response, KMH2 showed NOXA, HRK and BAD, L428 BIM and HRK, L1236 BIM and BAD, and DEV showed no response (Figure 4A). With the combination therapy, SUPHD1 showed additive effects in all curves with synergy in MCL-1i, KMH2 showed synergy with all 4 inhibitors and became sensitive to all 4 BH3 mimetics. L428 and L1236 showed most synergy in BCL-XLi and there was some effect in the combination of tamoxifen with venetoclax and navitoclax on DEV (Figure 4B and Supplemental Table S2). There was a clear additive response to tamoxifen when using BH3 mimetics. The effects were cell line-specific and dynamic BH3 profiling could predict responses well (Table 4).

## 3. Discussion

We set out to check expression of all anti-apoptotic proteins in HL and the sensitivity of HL cell lines to BH3 mimetics in combination therapy. We found that BCL-2 is expressed in 60% of cases, which is the lowest percentage in HL of the anti-apoptotic proteins tested; interestingly, BCL-2 is not expressed in NLPHL. Our numbers compare well with the paper from Rassidakis who reported 65% in NS and 47% in MC [14]. BCL-XL was reported to be between 67 and 94% [2,15] and BCL-W at 94% [3], both comparable to our results. MCL-1 was reported at 29% in one report, considerably lower than our results, but they considered cases positive only when at least 50% of HRS cells were positive [2]. We can conclude that in most cases at least 2 anti-apoptotic proteins are expressed in HL, and BCL-XL and BCL-W are the most commonly expressed.

For the sensitivity of the HL cell lines to BH3 mimetics, SUPHD1 is BCL-2 sensitive, L428 and DEV are BCL-XL sensitive, while KMH2 and L1236 are not sensitive. Recently it was described that KMH2 was sensitive to venetoclax [13] and ABT-737 or navitoclax (triple inhibitor) [3,7], SUPHD1 and L1236 to venetoclax [13], and L428 to BCL-XL and triple inhibitors, but not venetoclax [3]. The differences in sensitivity can be explained by the usage of higher doses like 5 or 10 µM for experiments, or daily 1 µM doses for 10 days, while we generally keep 1 µM as a limit for sensitivity. Comparable to the paper of Adams, we do see not a major role for MCL-1 in HL [3].

In the combination therapy with doxorubicin, we see a strong navitoclax and BCL-XLi response, except for L1236, which has a very limited response to doxorubicin. The triple inhibitor ABT-737 has been published to be synergic with doxorubicin in KMH2 [7], while an additive effect was shown with venetoclax and doxorubicin for KMH2 as well [13].

Like in other B cells, ERβ is expressed in Hodgkin lymphoma. Expression of ERβ was already shown in L428 in 2006 [10] and in a cohort of 27 pediatric HL patients in which 21 patients showed ERβ expression in more than 20% of HRS cells [11]. We observed a similar percentage of positive cases with a different antibody, albeit low levels of ERβ are seen in most cases.

The combination of tamoxifen with venetoclax was shown to be synergistic for ERβ positive breast cancer [16]. In HL cell lines, we see that the effect of tamoxifen is more diverse, affecting both BCL-2 and BCL-XL, and possibly BCL-W. Since navitoclax can inhibit BCL-2 as well as BCL-XL and BCL-W, the use of navitoclax would possibly be more advantageous. With tamoxifen, we could also sensitize cell lines that were not sensitive for BH3 mimetics to navitoclax and BCL-XL in the case of L1236 and to all 4 inhibitors in the case of KMH2. So, the addition of tamoxifen to BH3 mimetics could be beneficial in HL as well.

We see the strongest effect of doxorubicin with the BCL-XL inhibitor and navitoclax and think the combination of doxorubicin with navitoclax and also of tamoxifen with navitoclax could be effective treatment options in HL. In the cell lines, there is a limited effect of the BH3 mimetics alone, but we did observe an effect with combination therapy. The side-effects, especially thrombocytopenia, as well as the introduction of venetoclax as a specific BCL-2 inhibitor, have somewhat halted the introduction of navitoclax. The thrombocytopenia is shown to be reversible [17], and a continuous dosing schedule instead of an intermittent increased platelet numbers [18].

Given the current high cure rates with standard first line chemotherapy and radiotherapy in young HL, it would be logical to test effects of navitoclax and doxorubicin in patients who had multiple relapses or elderly that are unfit to tolerate more intensive treatment regimens. If effective, combined use of these drugs in the future might enable the elimination of toxic drugs that are used in first line treatment like bleomycin and procarbazine. The combination of tamoxifen and navitoclax is likely to be even less toxic and might serve as a treatment option for elderly of frail patients that have a dismal prognosis with current treatment options.

In conclusion, HL patients express multiple anti-apoptotic proteins in the HRS cells to protect them from apoptosis. In the cell lines, we are able to show that the combination therapy with a triple inhibitor as BH3 mimetic in combination with either tamoxifen or doxorubicin can be used as treatment in HL.

## 4. Materials and Methods

### 4.1. Cell Culture

The HL cell lines L1236 and KMH2 were cultured in RPMI1640 with 10% fetal bovine serum (FBS; HyClone Thermo Scientific, Waltham, MA, USA), 1% penicillin-streptomycin (Lonza BioWhittaker, Walkersville, MD, USA), and 1% glutamine (Lonza BioWhittaker). The HL cell line DEV was cultured in RPMI1640 with 20% FBS, and L428 was cultured in RPMI1640 supplemented with 5% FBS. Cell line SUPHD1 was cultured in McCoy’s medium supplemented with 20% FBS. All cell lines are cultured at 37˚C in 5% CO_2_ in a humidified chamber. They were cultured 3 times per week. The identity of the cell lines was checked periodically by STR profiling, and cell lines were regularly tested for *mycoplasma* infection.

### 4.2. Immunohistochemistry

Immunohistochemistry was performed with antibodies against BCL-2 (M0887, Dako, Glostrup, Denmark), BCL-XL (SC7195, Santa Cruz Biotechnology, Santa Cruz, CA, USA), BCL-W (4G12E6, Novus biologicals, Bio-Techne, UK), MCL-1 (A3534, Dako) and ERβ (CWK-F12, 1:160, Developmental Studies Hybridoma Bank, Iowa, USA) on paraffin-embedded tissue sections after antigen retrieval (pH 6,pH 9, pH 9, pH 6, and pH 9, respectively). Staining was visualized using HRP-labeled secondary antibodies (Dako) and 3,3′-diaminobenzidine (Sigma Aldrich, St Louis, MO, USA). Appropriate positive and negative controls were performed for each staining. The cases were stained and scored on a tissue micro-array (TMA). The TMA contained a total of 84 HL cases with 67 cases of NS HL, 7 cases of MC, 1 NOS HL, and 9 NLPHL. Patient material was acquired in accordance with international regulations and professional guidelines (the Declaration of Helsinki and the International Conference on Harmonization Guidelines for Good Clinical Practice). This project has been approved by the internal review board of the UMCG under RR#201800551.

### 4.3. Nanostring

A nCounter Custom Codeset consisting of capture and reporter probes was hybridized to 100 ng of RNA for 16 h at 65 °C. RNA samples were mixed with 4 µL capture probe-hybridization buffer mix and 1 µg of capture probes. After hybridization, the sample was loaded on a nCounter SPRINT Cartridge and then processed on the nCounter SPRINT™ Profiler.

Expression data were analyzed using Nanostring’s nSolver analysis software (version 3.0.). The raw counts were normalized against the geometric mean of internal positive controls to compensate for technical variability. Next, the data were normalized against the geometric mean of the housekeeping genes GAPDH, POLR2A, and WDR55.

### 4.4. Fluorescent FLTX1 Staining

Cytospins of cell lines were made, dried for 20 min, and fixed with 4% paraformaldehyde for 10 min. Mitochondrial staining was performed using TOMM20 (1:200) for 1 h and TRITC labeled secondary antibody (1:100) + AB serum for 30 min, followed by FLTX1 (50 µM, MedChemExpress, Bio-Connect, Netherlands) staining for 4 h and DAPI (1:1000) staining overnight at 4 °C.

### 4.5. Metabolic Activity (Resazurin) Assay

6 × 10^4^ cells were incubated with increasing concentrations of BH3-mimetics (venetoclax, navitoclax, S63845 (MCL-1inhibitor (MCLi)) and A1155463 (BCL-XLi) all from Selleckchem, VWR, PA, USA) in combination with tamoxifen (Selleckchem) for 48 h or doxorubicin (Selleckchem) for 72 h. AlamarBlue (Thermo Fisher Scientific) was added eight hours prior to read-out (extinction 560 nm, emission 590 nm). IC50 data were calculated with GraphPad Prism.

### 4.6. Western Blot

Cells were washed with ice cold PBS and lysed in RIPA buffer (50 mM Tris/150 mM NaCl/2.5 mM Na_2_EDTA/1% Triton X-100/0.5% sodium deoxycholate/0.1% SDS in dH_2_0) with 1% phenylmethanesulphonyl fluoride (PMSF) for 30–45 min on ice. Protein concentration was determined using the Pierce™ BCA Protein Assay Kit (#23227; Thermo Scientific). Lanes were loaded with 40 μg protein and electrophoresis and blotting were carried out according to standard protocols. Staining was performed with primary antibodies for BCL-2 (Abcam, Cambridge, UK), BCL-XL, BCL-W, MCL-1 (Cell Signaling Technology, Danvers, MA, USA), ERβ (PPZ0506, Invitrogen, Waltham, MA, USA), ERα (Abcam), and GAPDH (1:10.000; NB600-502, Novus Biologicals) overnight at 4 °C in 5% milk.

### 4.7. BH3 Profiling—Plate Method

Static and dynamic BH3 profiles of cell lines were performed as previously described by de Jong et al. [19], with static profiles reflecting the BH3 response of untreated cells and dynamic profiles the status after treatment. HL cell lines were treated with 15 (SUPHD1) or 20 µM (the remainder) tamoxifen for 18 h prior to readout. Peptide concentrations were 1 µM BAD, 10 µM HRK and NOXA, and 0.1 µM BIM. A 20% response or change in response was considered biological functional.

### 4.8. BH3 Profiling—Flow Cytometry Based Assay

Cell lines were incubated at 1.0 × 10^6^ cells/mL for 18 h with 0.5 µM doxorubicin in RPMI medium supplemented with 10% fetal bovine serum, 1% Penicillin-Streptomycin, and 1% glutamine. After incubation, cells were washed with PBS and incubated with a fixable viability dye (65-0864-14, Invitrogen) for 30 min at 4 °C. After staining, cells were washed with and suspended in MEB 90.15 M Mannitol, 0.01 M HEPES, 0.15 M KCl, 1 mMEGTA, 1 mM EDTA, 0.1%BSA, and 5 mM Succinate, pH7.5) at 3–5 × 106 cells/mL. Cells were incubated with peptides (10 µM BAD, 100 µM HRK and NOXA, 1 µM BIM) in 0.001% digitonin at room temperature for 60 min in the dark. The reaction was stopped and cells were fixed by addition of 4% formaldehyde (104005, Merck) in PBS for 10 min at room temperature in the dark. To neutralize the formaldehyde N2 buffer (1.7 M Tris, 1.25 M glycine, pH 9.1) was added for at least 5 min. Cells were stained overnight for intracellular cytochrome-c in PBS containing 1% BSA in PBS, 2% Tween 20 (P7949 Sigma Aldrich) and 1:400 Alexa Fluor 488 anti-cytochrome-c antibody (# 612308, BioLegend, San Diego, CA, USA) at 4 °C. Flow cytometry analysis was performed on a FACS (BD Biosciences). Data were normalized to PUMA2A (negative control) and alamethicin (positive control). The dynamic BH3 profile (Δ Cytochrome C) was calculated by subtracting the percentage treated Cyt C from percentage untreated Cyt C [20].

### 4.9. Statistical Analysis

Data were analyzed using GraphPad Prism (GraphPad Prism [version 8.4.2]; GraphPad Software) and tested for significant differences using a paired *t*-test. Combination index was calculated using observed/expected, results under 0.7 are considered antogonistic, between 0.7 and 1.3 are additive and over 1.3 are seen as synergistic. * indicates *p* ≤ 0.05, ** indicates *p* ≤ 0.01 and *** indicates *p* ≤ 0.001.

## Figures and Tables

**Table 1:** Immunohistochemistry results of Hodgkin lymphoma cases for anti-apoptotic proteins and ERβ.

	BCL-2+	BCL2-	BCL-XL+	BCL-XL-	BCL-W+	BCL-W-	MCL-1+	MCL-1-	Erβ+	Erβ-
NS *n* = 67	37	26	54	10	48	6	46	18	35	24
MC *n* = 7	4	3	6	1	5	0	7	0	5	2
NOS *n* = 1	1	0	1	0	1	0	1	0	1	0
NLP *n* = 9	0	9	9	1	7	1	9	0	7	2
missing	4		3		16		3		8	
%	54%	46%	88%	13%	90%	10%	78%	22%	63%	37%

**Table 2:** Overview of the results on the cell lines of expression of BH3 proteins and sensitivities for mimetics.

Cell Line	Protein Expression	BH3 Response	Expected Sensitivity	Venetoclax	Navitoclax	BCL-XLi	MCL-1i
SUPHD1	BCL-2, BCL-W, MCL-1	BIM, BAD	venetoclax, navitoclax	0.17	0.13		0.31
KMH2	BCL-2, BCL-W	BIM	none				
L428	BCL-XL	BAD, HRK	navitoclax, BCL-XLi		0.33	0.18	
L1236	BCL-XL, BCL-W	BAD	venetoclax, navitoclax		4.3		
DEV	BCL-XL, BCL-W, MCL-1	BIM	none		0.7	0.1	1.2

Green is sensitive, red is insensitive, with lighter shades of green and orange as indications of intermediate sensitivity.

**Table 3:** Overview of the effect of doxorubicin treatment on HL cell lines.

Cell Line		Doxorubicin					
	IC50 (µM)	BH3 Response	Expected Sensitivity	Venetoclax	Navitoclax	BCL-XLi	MCL-1i
SUPHD1	0.16	BAD	venetoclax navitoclax				
KMH2	0.37	HRK	navitoclax BCL-XLi				
L428	0.21	BAD	venetoclax navitoclax				
L1236	4.01	-	-				
DEV	0.24	HRK, BAD	venotoclax navitoclax BCL-XLi				

Green is synergy, orange is an additive effect and red is antagonism.

**Table 4:** Overview of the effect of tamoxifen on HL cell lines.

Cell Line		Tamoxifen					
	IC50 (µM)	BH3 Response	Expected Sensitivity	Venetoclax	Navitoclax	BCL-XLi	MCL-1i
SUPHD1	18	BAD	venetoclax navitoclax				
KMH2	25	NOXA, HRK, BAD	venetoclax navitoclax BCL-XLi MCL-1i				
L428	22	HRK	navitoclax BCL-XLi				
L1236	24	BAD	venetoclax navitoclax				
DEV	27	-	-				

Green is synergy, orange is an additive effect, and red is antagonism.

## Data Availability

Not applicable.

## Supplementary Materials

The following supporting information can be downloaded at: https://www.mdpi.com/article/10.3390/ijms232213751/s1.
