# Supplementary figures and images for: Navitoclax Most Promising BH3 Mimetic for Combination Therapy in Hodgkin Lymphoma
Source: Int J Mol Sci. 2022 Nov 9;23(22):13751. doi: 10.3390/ijms232213751 (PMC9699187; doi:10.3390/ijms232213751)

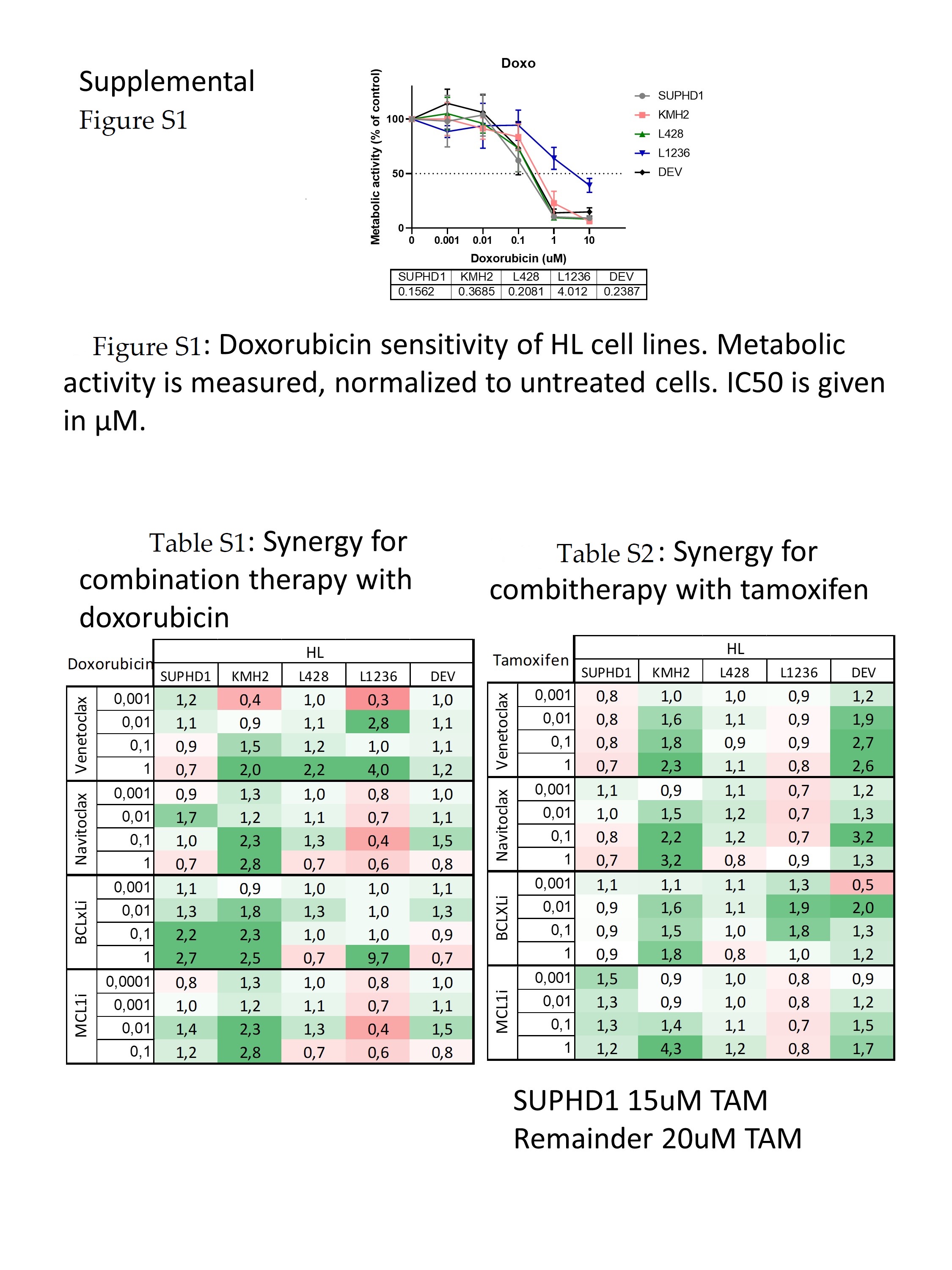

Supplement: Supplementary file 1 [file ijms-23-13751-s001.zip › ijms-2014657-supplementary.jpg]
